# Effect of Sound Amplification on Central Auditory Plasticity: Endbulb of Held as a Substrate

**DOI:** 10.3390/brainsci15080888

**Published:** 2025-08-20

**Authors:** Femi E. Ayeni, Michael A. Muniak, David K. Ryugo

**Affiliations:** 1 Hearing Research Unit, Garvan Institute of Medical Research, Sydney, NSW 2010, Australia; m@muniak.com (M.A.M.); david.ryugo@gmail.com (D.K.R.); 2 Nepean Clinical School, The University of Sydney, Sydney, NSW 2747, Australia; 3 St Vincent’s Clinical School, The University of Sydney, Sydney, NSW 2052, Australia; 4 Vollum Institute, Oregon Health & Science University, Portland, OR 97239, USA; 5 School of Biomedical Sciences, The University of Sydney, Sydney, NSW 2052, Australia; 6 Department of Otolaryngology, Head, Neck and Skull Base Surgery, St. Vincent’s Private Hospital, Darlinghurst, NSW 2010, Australia

**Keywords:** endbulb, sound amplification, sound enrichment, plasticity

## Abstract

Background: Hearing loss is known to cause structural and functional abnormalities in the central auditory pathways. Interventions with hearing aids that amplify acoustic signals have been developed to combat hearing loss. However, little is known about how such devices may affect the brain and mitigate the progression of hearing loss. We hypothesized that timely intervention that amplifies acoustic signals would delay further progression of hearing loss by maintaining central auditory activity and neural structure. Method: To that end, we provided eight weeks of acoustic stimulation tailored to compensate for subject-specific patterns of frequency loss in two mouse models of progressive hearing loss. We evaluated the effects of sound amplification on endbulb of Held anatomy at different ages of intervention in mice with early-onset (DBA/2) and late-onset (C57Bl/6) hearing loss. Results: We observed in both strains that endbulbs undergo rapid and progressive atrophy in untreated control subjects exposed to a baseline, unamplified, sound environment. In contrast, endbulb atrophy was significantly slowed in treated mice (*p* < 0.05). Conclusions: These data provide a possible explanation for how the brain benefits from sound amplification via hearing aid devices.

## 1. Introduction

The common symptoms of hearing loss are (1) a difficulty in understanding speech in noise, (2) distortions of loudness perception where small changes in loudness can cause severe pain and discomfort, and (3) the emergence of phantom sounds called tinnitus. These symptoms are not easily explained by damage to the inner ear, so evidence is building that the pathology is created by brain changes caused by auditory deprivation [1]. It is well established that sensory deprivation causes pathologic brain changes. Animal studies have shown striking changes in neuron health and axonal connections following visual, olfactory, somatosensory, and auditory deprivation (e.g., [2,3,4,5,6,7,8]). Auditory deprivation can manifest in somatic shrinkage [9,10,11,12], synaptic abnormalities [13] and abnormal physiological activity [14]. The clinical manifestation of this pathology is loss of sensitivity and perceptual distortions resulting from the structural and functional abnormalities in the central auditory pathway [15]. The pathology also causes an increase in the workload of the brain for addressing simple auditory tasks [16].

Early interventions of hearing loss have demonstrated that restoration of neural activity through cochlear implantation can help preserve structure and function in the auditory nervous system [17,18,19] and exhibit an impressive benefit for processing language [20]. This effect of cochlear implantation is hypothesized to result from the restoration of synapses and circuits in central auditory nuclei as shown for the cochlear nucleus [19], superior olivary complex [21], and auditory cortex [22]. Little is known, however, about how the treatment of hearing loss through sound amplification devices can affect central auditory structure.

There is considerable evidence that acoustic compensation of lost auditory input may boost neural activity [23,24,25], slow age-related hearing loss [26,27], and slow pathological changes in auditory structure [28]. These studies examined the effect of generous sound amplification regardless of hearing loss profile, as opposed to specifically compensating for frequencies with reduced sensitivity as practiced on humans [29,30]. One such study showed that exposing young DBA/2 mice (with early-onset hearing loss) or C57Bl/6 mice (with adult-onset hearing loss) to a controlled augmented acoustic environment at a level of 70 dB SPL could have an ameliorative effect on the degree of severity and progression of sensorineural hearing loss, as revealed by auditory brainstem response (ABR) thresholds, acoustic startle magnitude, and prepulse inhibition [23]. It is worth noting, however, that this study showed no further gain in auditory performance after 2–5 months of hearing loss onset, implying a narrow window of opportunity for mitigating the damage inflicted by hearing loss.

Thus, we investigated whether the restoration of auditory stimulation via sound amplification devices reversed or delayed the onset of central auditory system pathologies following hearing loss. To investigate the effect of sound amplification on central auditory structure, we studied a large auditory nerve ending in the cochlear nucleus called the endbulb of Held, which is found in all terrestrial vertebrates [31]. The central termination of an individual endbulb is featured by a characteristically large ending featuring several stout branches off the main axon that in turn branch repeatedly to give rise to a variable array of *en passant* and terminal endings [32,33,34]. Endbulbs of Held are known to play a significant role in auditory sound transmission [35,36] and have been implicated in the circuit that processes interaural timing information and sound localization cues [37,38,39].

The endbulb is well studied and known to exhibit activity-related changes in structure and function to both stimulation and deprivation [40,41,42,43,44]. In congenitally deaf cats, the endbulb exhibits a reduced branching pattern and hypertrophied synaptic structure compared to normal hearing cats [13,45,46]. Similarly, endbulb morphology is abnormal in mouse models of hearing loss (DBA/2 and Shaker-2) as compared to normal hearing CBA/Ca mice [41,47,48,49,50,51]. Thus, the endbulb can serve as a model for studying the structural effects of sound amplification.

Given high levels of brain plasticity [52,53], the existence of a sensitive period for intervention [54,55,56], and the dependence of brain structure on sensory experience [24,57], we sought to address the largely ignored but very important question of whether hearing aids that amplify the specific frequencies that are lost in hearing disorders also have effects on central auditory structures and/or on the progression of hearing loss itself. We quantified the effects of auditory restoration with respect to changes in endbulb of Held morphology as related to ABR thresholds.

## 2. Materials and Methods

### 2.1. Animals

**Figure 1:**
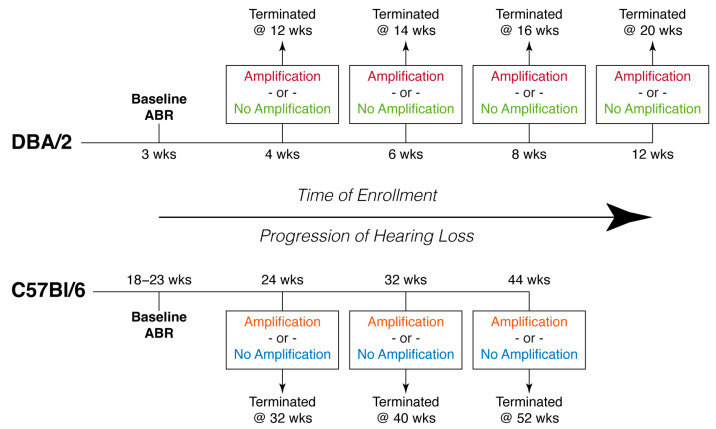
Schematic diagram showing cohorts and treatment timeline. All mice had a baseline auditory brainstem response recording at an age when they still retained their normal hearing profile. Mice were genetically predisposed to lose hearing as they age, with DBA/2 mice losing hearing at an early age, and C57Bl/6 mice experiencing loss in late adulthood. Each age cohort was age matched with CBA/Ca normal hearing mice. The experimental mice received eight weeks of treatment with amplified sound before termination. Each age cohort reflects a different severity of hearing loss (due to age-related progression) and a different time of intervention.

A total of 83 DBA/2, C57Bl/6, and CBA-GlyT2EGFP mice were used in this study. Each treatment and control group comprised 3–5 subjects. There were 20 C57Bl/6 mice (control = 11, enriched = 9) that formed three age cohorts: 24-, 32-, and 44-weeks enrolment. There were also 46 DBA/2 mice (control = 27, enriched = 19), which formed 4-, 6-, 8-, and 12-weeks enrolment. For the DBA/2 strain, there were also two additional cohorts: (1) a cohort that received extended stimulation (i.e., sound amplification) for 16 weeks from 4 weeks of age until termination at 20 weeks; and (2) a cohort raised as control in the animal vivarium (ambient sound level: 60–67 dB SPL (RMS) from 4 weeks until 52 weeks of age). Lastly, 17 CBA/Ca mice served as age matched controls for the different models of hearing loss, and were grouped into five age cohorts: 4, 8-, 12-, 32-, and 44-weeks enrolment. All animals received at least eight weeks of auditory stimulation before being terminated (Figure 1).

### 2.2. Auditory Brainstem Responses

Auditory brainstem responses (ABRs) were measured weekly from mice beginning at various age points until they were terminated. Mice were anaesthetized with 100 mg/kg ketamine and 20 mg/kg xylazine and placed on an infrared heating pad inside a sound-attenuating chamber (Sonora Technology, Gotenba, Japan). Ophthalmic ointment was applied to both eyes to prevent corneal irritation. ABRs were differentially recorded from the scalp using subcutaneous platinum needle electrodes that were positioned over the left bulla and at the vertex of the skull. A ground electrode was inserted into the muscle of the hind leg. Before recordings were made, the mouse’s head was positioned with its right pinna at 10 cm away from and at the same level as the free-field speaker (MF1; Tucker Davis Technologies [TDT], Alachua, FL, USA). Clicks and tone stimuli at 4, 8, 16, 24, 32, 40, and 48 kHz (5 ms duration, 0.5 ms rise/fall) were generated using a software-controlled signal processor (RZ6/BioSigRZ; TDT) and delivered at a rate of 10/s in 10-dB decremental steps from 90 dB to below any detectable threshold. Responses were amplified (RA16PA/RA4LI; TDT), bandpass filtered from 0.3–3 kHz, notched at 50 Hz, and averaged over 512 stimuli presentations (RZ6; TDT). Stored ABR wave forms were ported for analysis to a customised automated ABR analysis program, OpenABR (v0.2.0-aro; Samuel Kirkpatrick; https://doi.org/10.5281/zenodo.15629). Frequency threshold was defined as the lowest sound level that evoked a repeatable response waveform. All thresholds were determined by visual inspection of stacked waveforms, and most of these readings were independently verified by the software as the lowest stimulus level that evoked a response with an amplitude (peak-to-trough positions of Waves I–V) equal to or exceeding four standard deviations above the mean baseline noise level [58]. For a given frequency, a nominal threshold value of 110 dB SPL was assigned if there was no evoked response at the loudest stimulus intensity.

### 2.3. Experimental Treatment/Amplification

Both experimental and the control mice were housed in specially constructed, acoustically insulated sound booths, measuring 45 cm × 32 cm × 30 cm. Stimulation booths were kept in a separate room in the Garvan Institute’s animal care facility. Individual mouse cages with open-air wire mesh tops were placed inside each booth with ad libitum food and water. The presentation schedule of sound was computer controlled, passed through a hardware interface (ProFire 610; M-Audio, Cumberland, RI, USA), and amplified (SA1; TDT). Sound output was delivered from a calibrated high-frequency magnetic speaker (MF1; TDT) mounted on the top of each sound booth 26 cm from the floor of the mouse cage (Figure S1A).

The untreated control mice were subject to 24 h of ambient sound with an average level of 55 dB SPL (RMS). In contrast, to simulate hearing aid function, treated mice received sound amplification at a level commensurate to their frequency-specific threshold shift as determined by the weekly ABR audiogram for 8 weeks (Figure S1A). In other words, for each week it was under study, an individual mouse’s audiogram was compared to its initial baseline reading, and the value of any threshold shift (e.g., 10 dB loss @ 24 kHz) in the current audiogram was translated into an equivalent amount of extra amplification (e.g., 55 dB (baseline) + 10 dB (extra) SPL [RMS] @ 24 kHz) for the same frequency band in the sound stimulation it received over the following week. Amplification was effectively implemented by digitally altering the sound file delivered to the mouse that week using the graphic equalizer in Audacity (v2.0.6; http://audacity.sourceforge.net/)—the center frequencies of the equalizer bands were based on ABR tone stimuli. Frequencies for which the mouse did not have hearing loss were not amplified, and they continued to receive baseline sound at 55 dB SPL (RMS). The maximum stimulus level (baseline plus amplified sound) did not exceed 90 dB SPL to prevent any form of acoustic distortion or noise trauma. Thus, mice with threshold shifts > 35 dB only received the maximum stimulus level of 90 dB SPL at those frequencies. Additionally, mice with baseline ABR thresholds ≥ 90 dB SPL received maximum stimulation throughout the study. Sound stimuli were delivered from 7 pm until 7 am in the morning coinciding with their active nocturnal cycle.

The stimulation regimen consisted of natural sounds collected from the environment (e.g., dogs barking, bird calls, cat meows, animal room noise, mouse calls, and street noises) (Figure S1B). The sounds were digitized at a high sampling rate (192 kHz) to preserve high-frequency content in the mouse hearing range (D1000X; Pettersson Elektronik, Uppsala, Sweden) and patched into a continuous sound loop in Audacity (http://audacity.sourceforge.net/). In addition to these natural sounds, the sound stimuli also contained dynamic ripples that spanned 2–60 kHz, which were created using Matlab (vR2016a; Mathworks, Natick, MA, USA). Ripple stimuli are sounds that vary dynamically and randomly along the stimulus dimension in time, frequency, and intensity. Natural sounds and ripple stimuli were randomly interspersed together to prevent sound monotony and adaptation in mice. The presentation and duration of the sounds were automated and delivered randomly by the computer such that over the course of a 12-h shift, there was an equal amount of silence and stimulation, i.e., half the time in the 12-h period was quiet (50% duty cycle).

### 2.4. Cochlear Surgery and Auditory Nerve Injection

After their final ABR session, mice were subjected to cochlear surgery and auditory nerve injection to label auditory nerve fibers in the anteroventral cochlear nucleus (AVCN). Mice were anaesthetized using isoflurane (5% in 0.6 L/mm O_2_), which was maintained at 1.5–2% for the duration of the surgery. Ophthalmic ointment was applied to both eyes to prevent corneal irritation due to areflexia. In order to surgically expose the inner ear through a posterior auricular approach and to ensure that right ear was facing upward, the mouse was placed on its left lateral position. The mouse’s incisor was hooked to an anchor to hold its head in place and secured, and continuous anaesthesia was delivered through a nose cone. Under aseptic conditions, the right ear posterior auricular hairs were shaved, the skin swabbed with Betadine solution, and 0.01 mL bupivacaine applied around the exposed area. A light posterior auricular incision was made, the skin was raised, and the subcutaneous tissue along the cartilaginous external auditory canal was reflected downward until the bony bulla was reached. Using toothed forceps, the posterior inferior aspect of the bulla was carefully chipped away to increase the access and visibility of the otic capsule. At this junction, a blunt dissection was made into the cartilaginous external auditory canal to gain an access to the tympanic membrane (TM). The TM was punctured, and the malleus and incus removed. The stapedial artery was then cauterised with a low-temperature cautery (Medtronic, North Ryde, NSW, Australia) at its inferior and superior portion, followed by the removal of the stapes from its attachment to the oval window membrane. After the stapes had been removed, a fine-end curved hook was inserted into either the round or oval window to chip off the lateral wall of the otic capsule. The modiolus was located in the cochlea and 2–3 holes were made in its apical and middle turns with an acupuncture needle. A glass micropippete, 80–90 µm tip diameter, filled with 5% neurobiotin (SP-1120; Vector Laboratories, Burlingame, CA, USA), was used to pressure-inject 5 µL of neurobiotin into the modiolus of cochlea (Nanoject II; Drummond Scientific Company, Broomall, PA, USA). The pipette was withdrawn, and the animal allowed to survive for 4–6 h. Mice remained deeply anesthetized and received adequate analgesics according to the approved ethics protocol during the survival period.

### 2.5. Tissue Preparation

At the end of the survival time, animals were intraperitoneally injected with a lethal dose of sodium pentobarbitone (55 mg/kg). The thorax was dissected to expose the heart and 0.01 mL heparinized saline was injected directly into the right ventricle to prevent occlusion of the cardiac blood vessels. A slight cut was made at the right atrium with scissors, which was immediately followed by perfusing with 4 mL of 1% sodium nitrite prewash in 0.1 M phosphate-buffered saline and then 60 mL of 4% paraformaldehyde in 0.1 M phosphate buffer. The fixative solution was pushed through for a period of 10 min. The mouse head was postfixed overnight at room temperature in 4% paraformaldehyde on a shaking platform. The next day, the brain was dissected from the skull, blocked, and embedded it in a gelatin-albumin mixture hardened with glutaraldehyde. The gelatin-albumin block was trimmed with a razor blade, mounted, and cut into 50 µm coronal sections with a vibrating microtome (VT1200S; Leica Systems, Nussloch, Germany). Free-floating tissue sections were collected in 0.12 M Tris buffer (TBS) and then rinsed three times in 0.12 M TBS within 5 min. Thereafter, tissue sections were incubated in fluorescent Streptavidin, Alexa Fluor 568 conjugate (1:1000; Cat # S11226; Invitrogen, Waltham, MA, USA) in 0.12 M TBS buffer for one hour to visualize neurobiotin filled endbulbs of Held in the AVCN. After incubation, tissue sections were rinsed thrice in 0.12 M TBS for 5 min, mounted on microscope slides, and coverslipped using Vectashield (H-1400; Vector Labs, Burlingame, CA, USA). Slides from all mouse cohorts were mixed and coded by a neutral party so that the observer was blinded to the ages of the subjects during analysis.

### 2.6. Confocal Image Acquisition and Analysis

Z-stack images of coded tissue sections were acquired with a confocal microscope (DMI 6000 SP8; Leica Systems; 63×/1.20 water immersion objective, NA 1.2, pinhole 1 Airy unit), using 561 nm (intensity: 15%) laser excitation, at a resolution of 1024 × 1024 pixels and a z-step size of 0.35 µm, and a setting of one frame and line averaging. Only endbulbs with an attached axon were imaged. Both low- and high-frequency zones of the AVCN were sampled with each endbulb surrounding the silhouette of the somata of a bushy cell. The confocal parameters for image acquisition were kept constant for all cases. After image acquisition, the stacks of endbulb slices were ported to Imaris software (v7.2; Bitplane, Zurich, Switzerland). This software allows the visualization of complex structures in 3-dimensional space based on fluorescence intensity and size [59]. In this study, no image processing was done other than median filtering. To quantify endbulb surface area and volume, we first removed background noise using a median filter. The same median filter (3 × 3 × 3) was applied to all images since it does not alter the edge of an image [60]. Next, a solid surface of an endbulb was created in the “surpass view”, followed by the delineation of a segment of interest in the image plane. We disabled the “smoothing” tool on the software as this caused artificial uniformity to a cell surface, but we enabled the background subtraction option [59]. The software automatically determined the minimum diameter of the endbulb structure. This procedure was followed by manual thresholding of the endbulb fluorescence signal and applying a threshold setting that was low enough to completely detect the endbulb of interest and its fine details. Following thresholding, the software allowed us to delineate each auditory nerve axon (grey) from its terminal endings (red) as shown in Figure S2. The axon leading to the endbulb terminal was cut off and the ending quantified by measuring its surface area and volume. The observer was blinded to all slides from image acquisition through analysis. Further consideration was that the degree of curvature and swellings in each endbulb may determine the level of its complexity. In order to evaluate any change in complexity of endbulb morphology in response to treatment, we calculated the surface area to volume ratio and shape factor for each endbulb. The shape factor is a dimensionless quantity that describes the shape of a particle independent of its size and is calculated as (SA1.5)/(6πV), where SA and V are the endbulb surface area and volume, respectively [61]. A shape factor of 1.0 represents a perfect sphere. A large shape factor or surface area to volume ratio typifies a complex endbulb.

### 2.7. Statistical Analyses

Each age cohort has 3–5 sound amplified and 3–5 control subjects. In general, data from individual animals in each age group were pooled together. Unpaired t test with Welch correction was performed for the ABR threshold analysis. Endbulb data were not normally distributed as determined using D’Agostino & Pearson omnibus normality test and Shapiro–Wilk normality test. Comparisons were made between treated and untreated groups at each age point using either the Mann–Whitney U Test, or, for comparisons with more than two groups, the Kruskal–Wallis one-way ANOVA with Dunn’s multiple comparisons post hoc test. Statistical tests were carried out in Matlab (vR2016a; Mathworks, Natick, MA, USA) or Prism (v.6; GraphPad, La Jolla, CA, USA) software. All error bars correspond to the standard deviation (SD). Statistical significance was accepted at *p* < 0.05.

## 3. Results

### 3.1. Sound Amplification and Threshold Change: DBA/2 Mice

**Figure 2:**
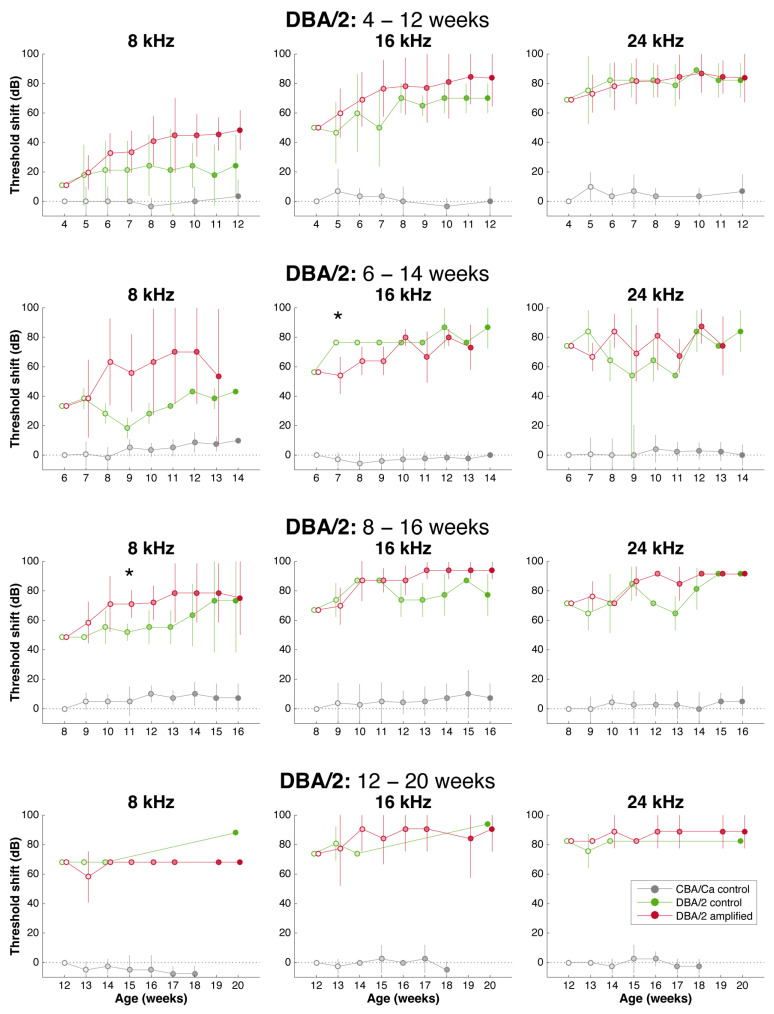
Mean shift in individual ABR thresholds over 8 weeks for DBA/2 mice during the stimulation paradigm. The shift in the ABR threshold for each animal relative to the threshold obtained at the time of enrollment was calculated at each age and then averaged. To illustrate how hearing levels may differ from “normal hearing” at the time of enrollment, these values were further offset by the difference between the mean threshold of DBA/2 mice at the age of enrollment and that of age-matched CBA mice. Comparative data for age-matched CBA mice (grey) are also shown. Rows from top to bottom show results from cohorts enrolled at 4-, 6-, 8-, and 12-weeks-of-age. Columns from left to right show results at 8, 16, and 24 kHz. Generally, both treated (red) and untreated (green) animals fared similarly. However, at 8 kHz for 4-, 6-, and 8-week cohorts, treated animals appeared to lose hearing at a faster rate than control animals, but this was generally not significant, where * indicates *p* < 0.05.

Baseline hearing thresholds were obtained from DBA/2 mice starting at 3 weeks of age. Even at this early age, some of the mice already had either severely elevated thresholds or no response to tones at 24, 32, 40, and 48 kHz (Figure 2).

The variability in thresholds for DBA/2 mice was striking. We began the enriched stimulation program for the early treatment cohort at 4 weeks of age. Due to the rapid pace of hearing loss, subsequent enrollments were made at brief intervals starting at 6, 8, and 12 weeks of age. Our results showed that hearing thresholds were markedly variable and deteriorated with age and progression of hearing loss (Figure 2). The subjects that received an enriched acoustic environment lost hearing at the same rate as the control mice. But when amplification treatment was delayed until 12 weeks of age, mice had no evoked response, and this status remained unchanged until the end of the treatment (Figure 2). The progression of hearing loss was so rapid and profound that neither early nor late acoustic amplification had any impact on the progress of hearing loss. The age-matched CBA mice have ABR thresholds that are 50–60 dB better for this cohort.

**Figure 3:**
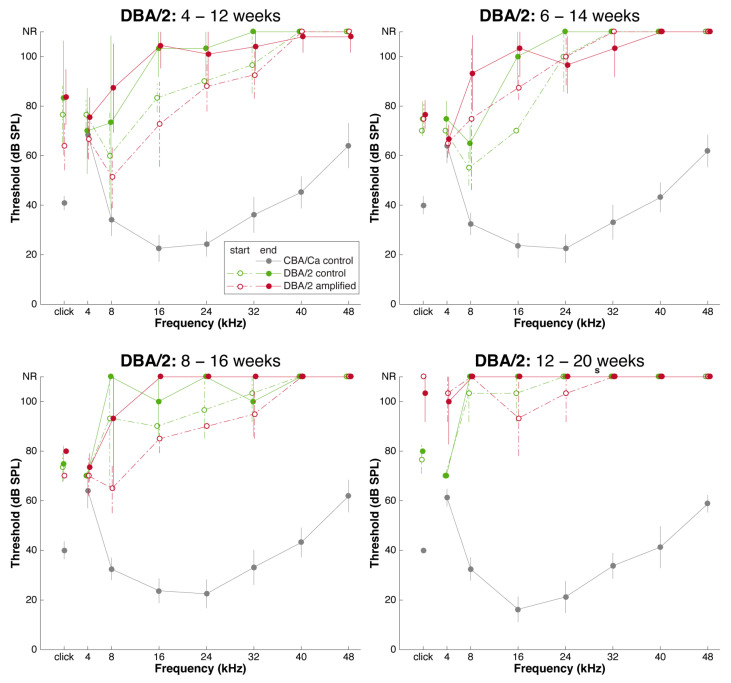
Average ABR audiograms before and after sound amplification in DBA/2 mice with juvenile-onset hearing loss. Audiograms are shown for cohorts that were enrolled beginning at 4-, 6-, 8-, and 12-weeks-of-age. After eight weeks of amplification, there appears to be no improvement in the hearing threshold of experimental (amplified) mice over controls. The high-frequency hearing loss was profound by 4-weeks-of-age and remained degenerate despite early treatment. Experimental mice in the late treatment group (12 weeks) were deaf at all frequencies by the time they were enrolled.

Stimulation had no clear restorative or protective effect for DBA/2 mice (Figure 2), a situation that reminded us of the power of genes. From these data, we plotted the mean shift in individual ABR thresholds over the 8 weeks of amplified stimulation (Figure 3). The shift in the threshold for each animal relative to the threshold recorded at the time of enrollment into the stimulation treatment session was calculated, averaged and compared with the control using unpaired t-test. Analysis did not reveal any significant difference between the ABR thresholds of mice that received sound amplification and the control mice *p* > 0.05 (Figure 2 and Figure 3). The thresholds for amplified cohorts and for control cohorts were similar: both groups lost hearing at a similar rate and with similar severity. These data are also compared to thresholds for CBA mice at comparable ages.

### 3.2. Sound Amplification and Threshold Change: C57Bl/6 Mice

Age-related hearing loss (presbycusis) has been modeled by C57Bl/6 mice in that they begin to lose high frequencies at around 6 months of age and this loss becomes progressively more severe to include even the low frequencies by about 12 months of age [62,63]. Similar to DBA/2 mice, we collected ABR tone thresholds on a weekly basis for C57 mice from 20 weeks of age. As can be seen, sensitivity to tones below 16 kHz remained steady until 24 weeks of age (Figure S3). After this time, thresholds in C57Bl/6 mice to higher frequency tones began to climb relative to those of CBA mice. This pattern of age-related high frequency loss has been reported before and verified that our strain was typical in terms of its hearing.

**Figure 4:**
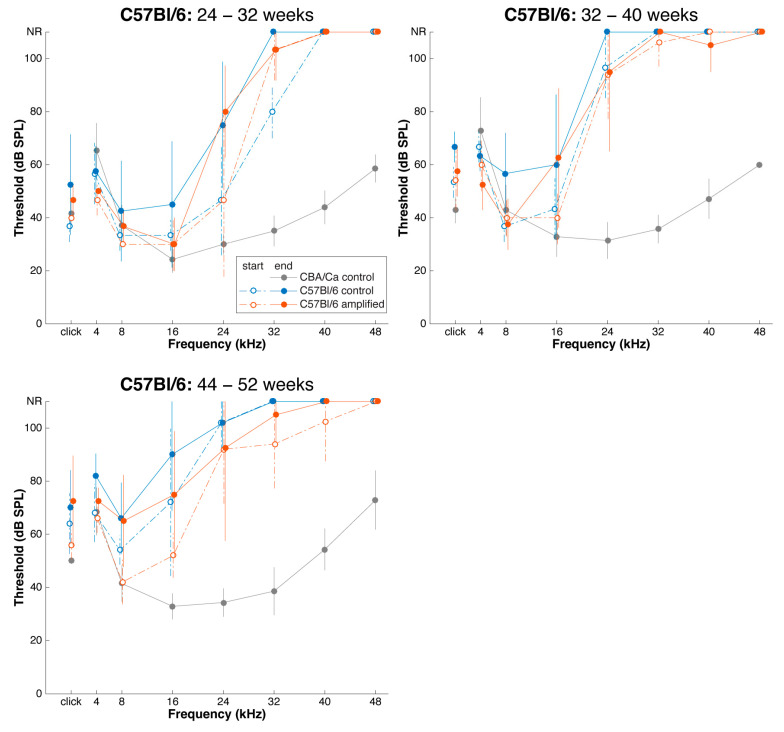
Average ABR audiograms before and after sound amplification in C57Bl/6 mice with adult-onset hearing loss. Audiograms are shown for cohorts that were enrolled beginning at 24-, 32-, and 44-weeks-of-age. After eight weeks of amplification, thresholds at 16 kHz and 8 kHz appear to be unchanged if animals are enrolled at 24 weeks and 32 weeks, respectively. High-frequency hearing loss was already profound by 24 weeks of age in all study groups and remained irremediable despite sound amplification. However, it is noteworthy that decay of thresholds for 8 and 16 kHz appeared to be delayed by the amplified auditory environment, although the result was not significant.

**Figure 5:**
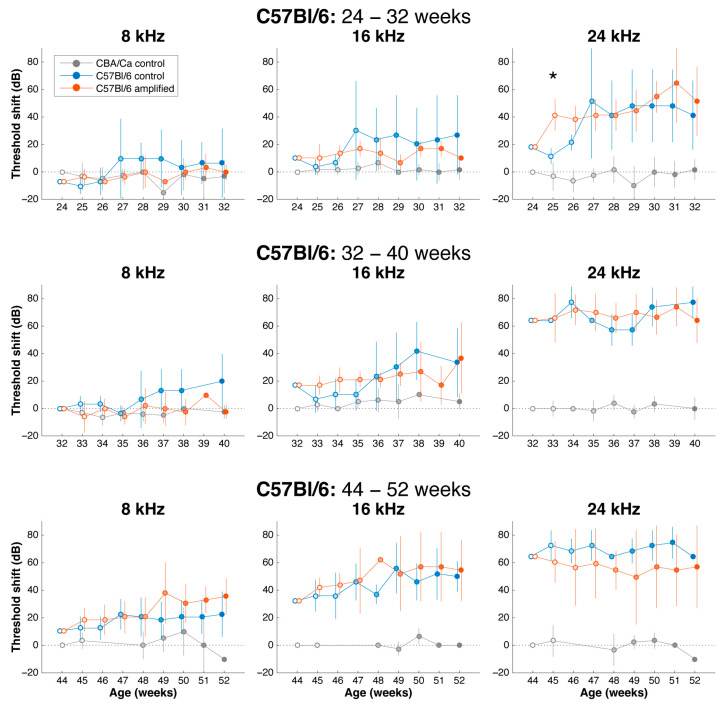
Mean shift in individual ABR thresholds over 8 weeks for C57Bl/6 mice during the stimulation paradigm. The shift in the ABR threshold for each animal relative to the threshold obtained at the time of enrollment was calculated at each age and then averaged. To illustrate how hearing levels may differ from “normal hearing” at the time of enrollment, these values were further offset by the difference between the mean threshold of C57Bl/6 mice at the age of enrollment and that of age-matched CBA mice. Comparative data for age-matched CBA mice (grey) are also shown. Rows from top to bottom show results from cohorts enrolled at 24-, 32-, and 44-weeks-of-age. Columns from left to right show results at 8, 16, and 24 kHz. Treated (red) animals tended to exhibit less dramatic increases in thresholds during the stimulation paradigm compared to untreated (blue) animals, particularly when enrolled at 24 or 32 weeks, although this was not significant. An asterisk (*) indicates *p* < 0.05.

C57Bl/6 mice were thus studied at 24, 32, and 44 weeks of age, with experimental cohorts receiving amplified stimulation for those frequencies that exhibited loss, and control cohorts receiving no extra amplification in the acoustic booth. The amplified environment appeared to shift the audiogram of the stimulated mice to the right, suggesting it delayed the progressive decline of auditory thresholds (Figure 4; cohort 32–40 weeks and 44–52 weeks). ABR audiograms collected before and after sound amplification revealed that thresholds at 8 kHz, 16 kHz, and 24 kHz were generally unchanged, although higher frequencies already exhibited profound loss by this time (Figure 5).

The shifts in the auditory thresholds over time are shown for 24, 32, and 44-week cohorts (Figure 5). Unpaired t-test analysis did not reveal any significant difference between the mice that received sound amplification and control., *p* > 0.05. Generally, due to small sample sizes, there was large variability in the data for ABR responses. These small sample sizes occurred because as animals aged, some died of unknown causes. Mice that completed their 8 week course of study were euthanized and their brains collected so that we had an age-graded series of histological preparations.

### 3.3. Effects of Sound Amplification on Endbulbs of Held: DBA/2 Mice

**Figure 6:**
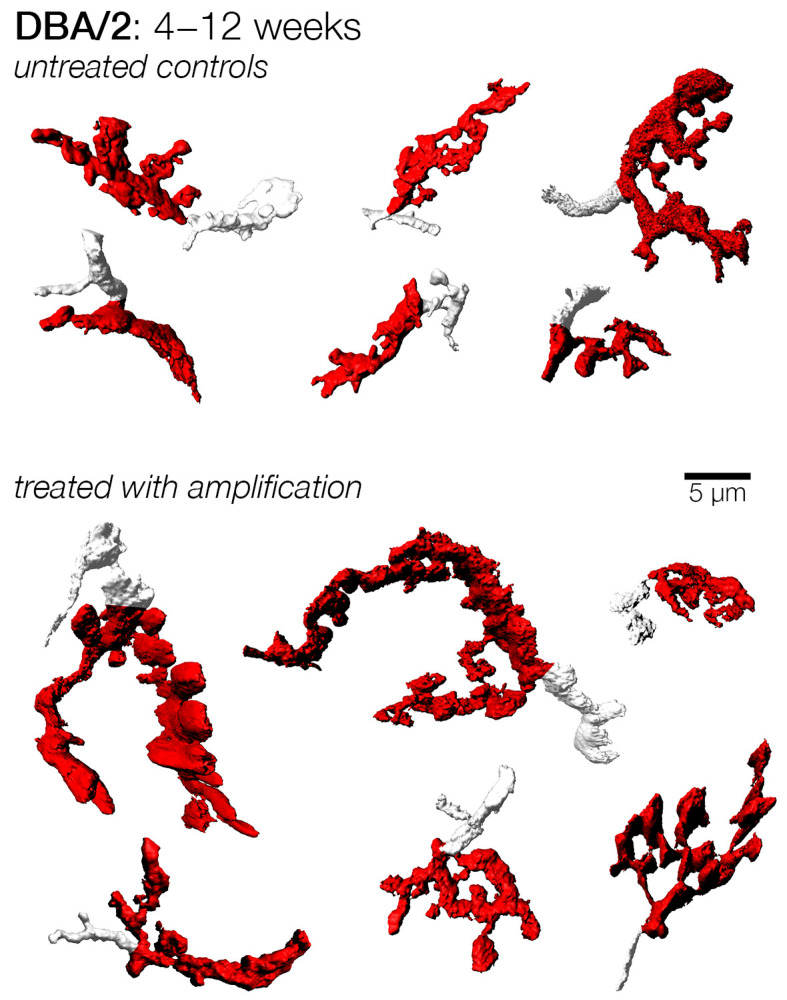
Examples of endbulbs from DBA/2 mice with and without early amplification from 4 to 12 weeks of age. DBA/2 mice exhibit a rapid progression of hearing loss. Endbulbs from mice treated (amplified) early relative to the onset of hearing loss were larger than untreated endbulbs, having more extensive branching. This result suggests that treated mice maintained their endbulb complexity and structure when acoustic input was restored. Untreated endbulbs appeared smaller and had less branching, suggesting a loss of the complex morphology normally attributed to endbulbs.

**Figure 7:**
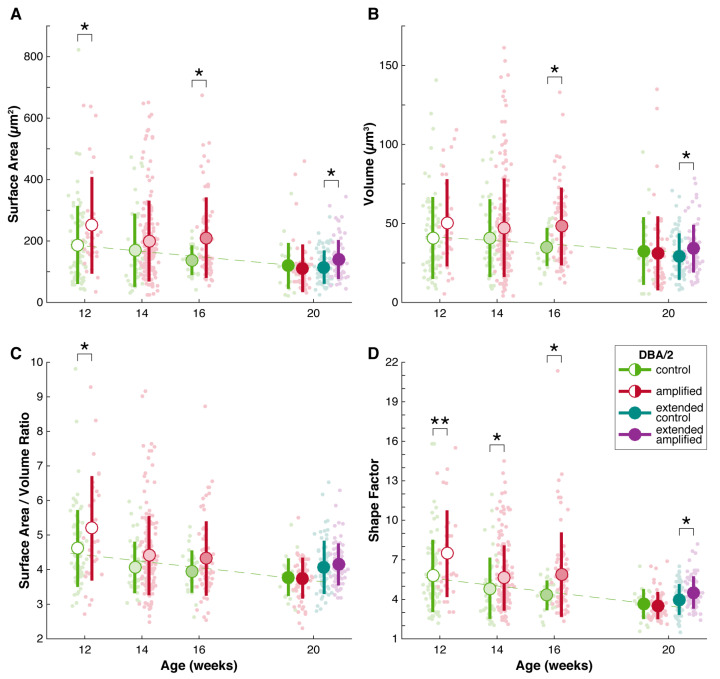
Quantification of endbulb morphology for DBA/2 mice with and without amplification enrolled at various ages. (**A**). Endbulb surface area declined with the progression of hearing loss in untreated mice (green; linear regression R2 = 0.94; *p* < 0.05). Early intervention with sound amplification treatment (red/purple) appeared to significantly slow down this reduction, but only if mice were enrolled at earlier ages. (**B**). Endbulb volume also declined with the progression of hearing loss (linear regression R2 = 0.89; *p* = 0.06). Amplification produced larger endbulbs in all but the 12–20 week cohort. (**C**). The surface area/volume ratio showed a moderate reduction with the progression of hearing loss (linear regression R2 = 0.77; *p* = 0.12). A significantly larger ratio value was observed for early treated endbulbs. (**D**). Shape factor is another indicator of complexity that relates object surface area to volume. With progressive hearing loss, endbulb shape factor reduced (linear regression R2 = 0.93; *p* < 0.05). Sound amplification therapy produced significantly more complex endbulbs (larger shape factor) for all treated groups except those enrolled at a late age (12 weeks). Vertical bars indicate mean ± standard deviation. * = *p* < 0.05; ** = *p* < 0.01. Green, untreated cohorts; red/purple, treated cohorts (8/16 weeks amplification).

Having established that sound enrichment had little or no influence on the progressive decline of auditory thresholds, we sought to investigate if sound amplification had any effect on the central auditory system, with a focus on the morphology of endbulb of Held. As reported earlier, early treatment was commenced at 4 weeks and later treatments were set at 2-week intervals. We tailored treatment to the individual subject by using their baseline hearing threshold that was obtained at three weeks as a reference “best” hearing threshold, over which sound compensation was given. Then, after eight weeks of continuous sound compensation, we examined the endbulbs of Held of each mouse. For the earliest treatment cohort that was enrolled at 4 weeks of age and stimulated for 8 weeks, the endbulbs collected at 12 weeks from control subjects appeared smaller than those of amplified subjects (Figure 6). Indeed, the surface area of endbulbs from control subjects was significantly smaller by 25% as compared to the enriched endbulbs (control: 187.0 ± 127.0, n = 75; Amplified: 250.9 ± 157.3, n = 33; *p* < 0.05). Endbulb volume was also reduced by 20% in control subjects, and both metrics of complexity—surface area to volume ratio and shape factor—showed that endbulbs from treated subjects were significantly more complex (Table 1; Figure 7). The enriched endbulbs showed more elaborate branching and more terminal swellings, whereas sound deprived endbulbs appeared smaller, shrunken, and less complicated (Figure 6). Although there were still some endbulbs that were large and complex in the control cohort, such occurrences were infrequent.

The next cohort was enrolled at 6 weeks and stimulated until they were 14 weeks of age. Mice in this cohort had only experienced three weeks of hearing loss from the time the baseline hearing threshold was obtained. After eight weeks of acoustic stimulation, amplified endbulbs appeared visually more complex in shape compared to untreated controls. The amplified endbulbs were approximately 16% larger both with respect to the mean surface area and volume, although these differences were not statistically significant (Table 1; Figure 7). Treated endbulbs, however, had a significantly larger shape factor value, indicating they were more complex following amplification (control: 4.84 ± 2.32, n = 44; amplified: 5.62 ± 2.47, n = 168; *p* < 0.05).

**Figure 8:**
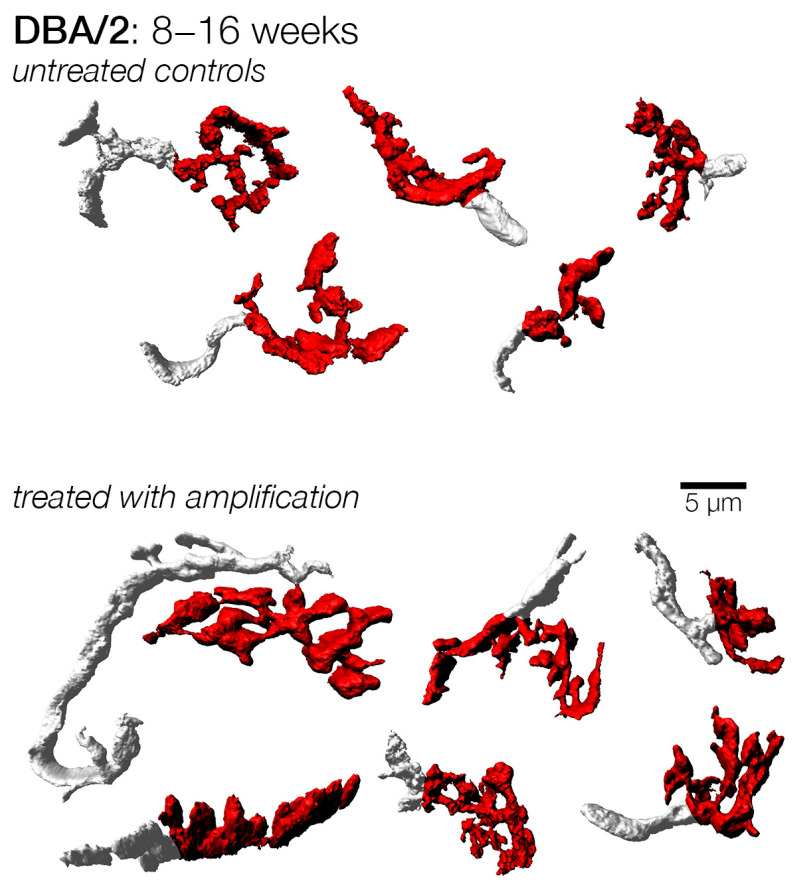
Examples of endbulbs from DBA/2 mice with and without mid-late amplification from 8 to 16 weeks of age. Endbulbs from mice treated (amplified) at a mid-to-late period of their hearing loss still appeared larger than untreated endbulbs, having more extensive branching. This suggests that treated mice maintained their endbulb complexity and structure even when acoustic input was slightly delayed relative to the onset of progressive loss. Untreated endbulbs appeared smaller and had less branching, suggesting a loss of the complex morphology normally attributed to endbulbs.

A third set of mice were subject to a more prolonged duration of hearing loss, and these were enrolled at 8 weeks. Similar to the cohorts enrolled at earlier ages, endbulbs from subjects that did not receive amplification appeared more stunted in appearance (Figure 8). Indeed, both the mean endbulb surface area and volume were significantly larger in treated subjects, exhibiting 53% and 37% increases, respectively. This change was also reflected in a significantly larger shape factor (Table 1; Figure 7).

**Figure 9:**
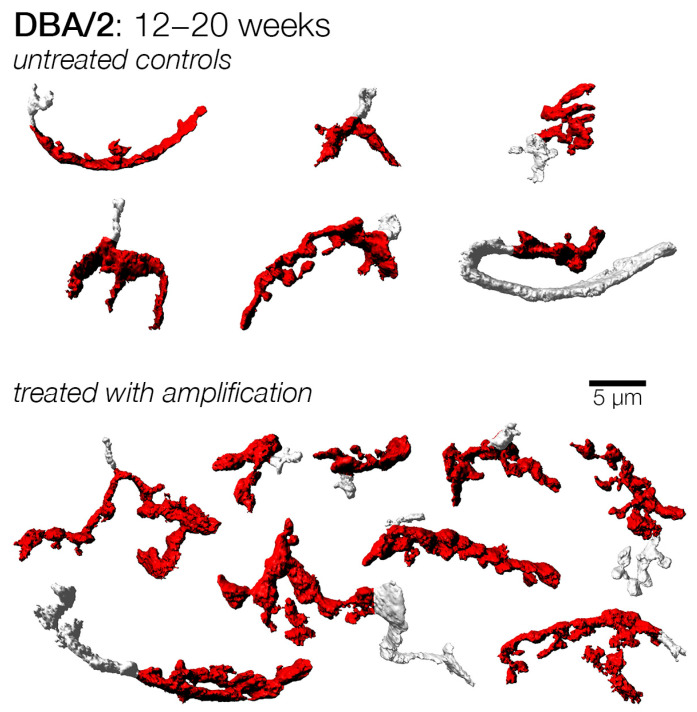
Examples of endbulbs from DBA/2 mice with and without late amplification from 12 to 20 weeks of age. Endbulbs from mice with delayed enrollment relative to the onset of progressive hearing loss appeared smaller than younger cohorts, regardless of whether they received amplification or not. At the time of enrollment, some mice showed no evoked responses in most frequencies tested—if they still retained residual hearing at low frequencies, they did so at an elevated threshold. Thus, a significant delay in the treatment of hearing loss via sound amplification therapy appears to affect the potential to rescue endbulb morphology in DBA/2 mice.

We delayed the initiation of treatment to 12 weeks of age for another cohort. At this age, most subjects in the treatment group had no evoked response to sound tone (Figure 2). Meanwhile, acoustic input that reflected the loss was also provided to the enriched subjects for eight weeks, while the control subjects received no sound amplification. Endbulbs in both cohorts appeared quite atrophied (Figure 9), and morphometric analyses did not reveal any significant differences (Table 1; Figure 7). Therefore, it appeared that sound compensation only has an effect if the treatment is initiated prior to 12 weeks of age in DBA/2 mice, before hearing loss becomes severe.

### 3.4. Endbulbs of Held with Extended Duration of Amplification: DBA/2 Mice

**Figure 10:**
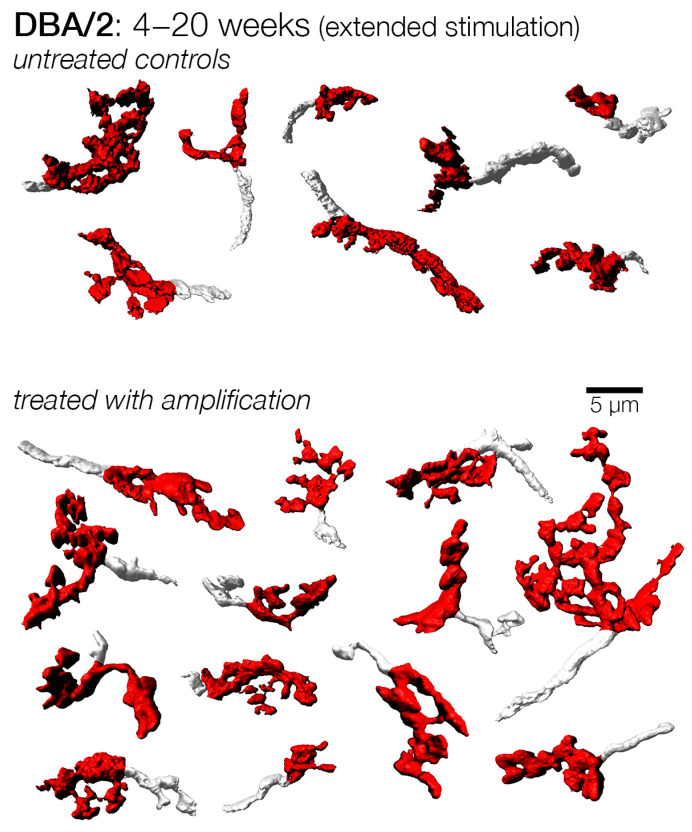
Examples of endbulbs from DBA/2 mice with and without early-extended amplification from 4 to 20 weeks of age. Endbulbs from mice without treatment appeared small and atrophied, similar to those from mice enrolled from 12–20 weeks. In contrast, endbulbs from mice that received extended amplification treatment from 4 weeks all the way until 20 weeks appeared significantly larger and more complex. Despite this apparent preservation of endbulb morphology, these endbulbs still appeared somewhat smaller than those of treated animals that were examined at earlier time points (12, 14, and 16 weeks). Thus, while sound amplification therapy may preserve endbulb morphology relative to age-matched controls, there is still a degree of atrophy in endbulbs of DBA/2 mice that was not possible to prevent.

We investigated the effect of sound amplification with another experiment that combined early treatment with late termination to examine if the duration of treatment is an important factor for consideration in treating hearing loss. In this experiment, control and sound amplified cohorts were enrolled at 4 weeks of age, the treated group received extended sound compensation until 20 weeks of age, whereas the control group continued to receive baseline stimulation for the same duration. We examined endbulbs from these groups at 20 weeks of age, and found that, while endbulbs from control subjects resembled those from the late-treatment cohort, endbulbs from treated subjects exhibited a more complex shape (Figure 10). Indeed, while endbulbs from both the extended treatment and untreated cohorts were smaller than those examined at earlier ages, treatment nonetheless resulted in significantly larger mean endbulb surface area and volume, as well as shape factor (Table 1; Figure 7). This observation suggests that early treatment has a more robust effect than later treatment and is consistent with the idea that young animals are more responsive to stimulation treatment. We also examined endbulbs from DBA/2 mice that were left alone up to one year of age with no treatment. These endbulbs were strikingly atrophic with few branches and obliterated complexity (Figure S4).

### 3.5. Effects of Sound Amplification on Endbulbs of Held: C57Bl/6 Mice

**Figure 11:**
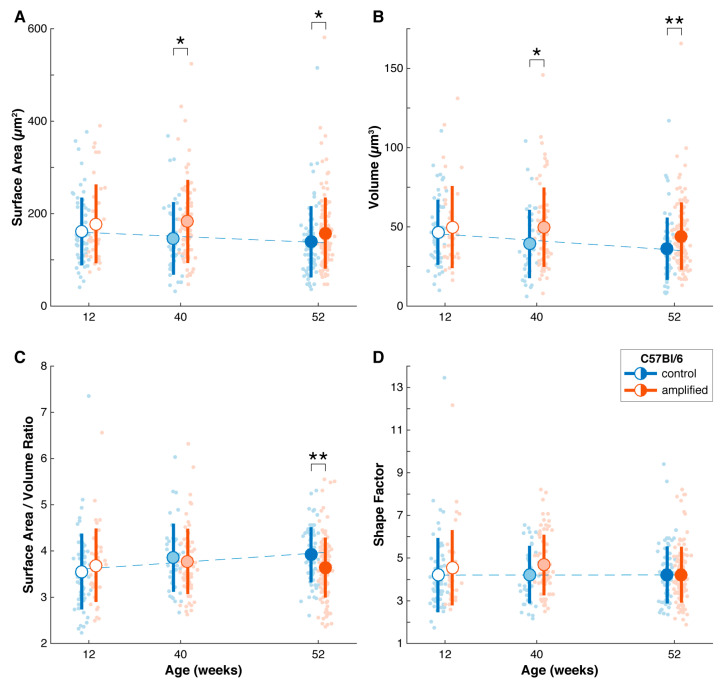
Quantification of endbulb morphology for C57Bl/6 mice with and without amplification enrolled at various ages. (**A**). Endbulb surface area only somewhat declined with the progression of hearing loss in untreated mice (blue; linear regression R2 = 0.91; *p* = 0.20). Intervention with sound amplification treatment (red) appeared to result in slightly larger surface areas. (**B**). Endbulb volume declined with the progression of hearing loss (linear regression R2 = 0.88; *p* = 0.23). Amplification produced larger endbulbs, especially in the older cohorts. (**C**). Curiously, the surface area/volume ratio showed a moderate increase with the progression of hearing loss (linear regression R2 = 0.79; *p* = 0.30). Treated endbulbs only differed for late treatment. (**D**). Shape factor is generally a more reliable indicator of object complexity. With progressive hearing loss in C57Bl/6 mice, there appeared to be no dramatic change in endbulb shape factor (linear regression R2 = 0.08; *p* = 0.82). Sound amplification therapy did not appear to alter endbulb complexity regardless of the age of enrollment. Vertical bars indicate mean ± standard deviation. * = *p* < 0.05; ** = *p* < 0.01. Blue, untreated cohorts; red, treated cohorts.

C57Bl/6 mice with adult-onset, slow progressive hearing loss was examined. All mice had a baseline hearing assessment at 18–23 weeks when they still retained their normal hearing, providing a reference for hearing loss. The data obtained from this strain followed a consistent theme with the data obtained in DBA/2 strain except for a shift in the timeline. Early treatment commenced at 24 weeks and animals were monitored until 32 weeks. Endbulbs from both study groups were examined at this age. Our result showed that endbulbs did not significantly change between the amplified and untreated mice for this age cohort. Measures of mean endbulb surface area and volume showed slight but statistically insignificant increases following treatment, *p* > 0.05 (Table 2; Figure 11). This finding suggested that endbulb atrophy from acoustic deprivation had not become pronounced in the control mice. We next examined endbulbs from mice that had a much longer duration of hearing loss, with enrollment made at 32 weeks and monitored until 40 weeks. Our data showed that both the mean endbulb surface area and volume of control mice were significantly reduced as compared to the amplified mice (Table 2; Figure 11). These differences, however, were not reflected in measures of endbulb complexity (Endbulb shape factor, *p* = 0.82).

**Figure 12:**
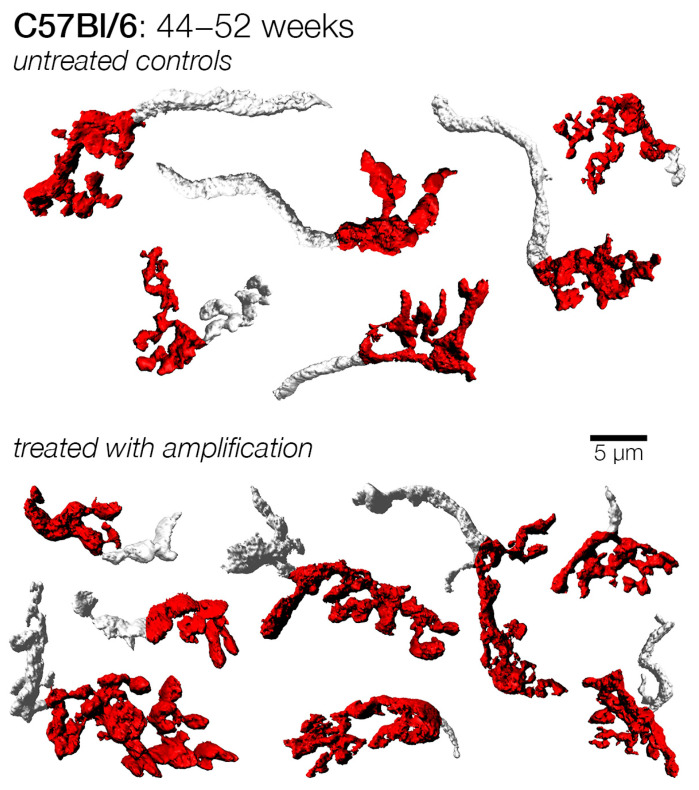
Examples of endbulbs from C57Bl/6 mice with and without late amplification from 44 to 52 weeks of age. Endbulbs from both treated and untreated cohort at 1 year retained a similar degree of branching and complexity. Endbulbs from treated (amplified) mice, however, tended to be larger in overall size.

The latest enrollment was made at 44 weeks of age. Mice in this cohort had experienced a much longer duration of hearing loss given the age at enrolment. They were stimulated until they were 52 weeks of age (Figure 12). We found that the mean endbulb surface area and volume were significantly larger for the amplified mice in this group (Table 2; Figure 12). Curiously, despite these increases, the surface area to volume ratio showed a significant reduction in endbulb complexity for the amplified subjects, and no change in shape factor (Table 2). Endbulbs from the C57Bl/6 strain showed structural enhancement in terms of surface area and volume as a result of amplification. They did not, however, reveal any significant change between the enriched and untreated age cohorts with respect to shape factor (Figure 11). The lack of a statistically significant difference in endbulb complexity may be because endbulb atrophy in the unamplified C57Bl/6 strain was not as severe as compared to that of unamplified DBA/2 mice. The data also emphasize the importance of strain differences when comparing the outcomes of experimental treatments.

## 4. Discussion

### 4.1. Threshold Change

Auditory threshold measurements reflect hair cell health whose activity is functionally driving auditory nerve responses [64,65]. Our data suggest that auditory thresholds may be partially rescued in moderate hearing loss, mainly in the mid-low frequency region as observed in C57Bl/6 mice. We did not see any significant change in the threshold of treated DBA/2 mice. Despite early intervention, hearing loss still progressed rapidly in this strain, such that the treated cohorts were no different from the control groups. It seems the aggressive genetic mutation [66] that rapidly destroys the hair cells made sound amplification therapy ineffective. This finding underscores the fact that the beneficial outcome of hearing aids is completely dependent on functional hair cells and is consistent with the findings of Turner and Willott [23] who reported that exposure of DBA/2 mice to auditory enrichment after 45 days of age did not yield any effect on hearing function.

In contrast, we observed a maintenance in the thresholds of early treated C57Bl/6 mice at 8 and 16 kHz. The most profound effect of threshold preservation occurred at frequencies that were presumably still normal during treatment since hearing loss progresses from the base (high frequency) to the apex (low frequency) in the cochlea. These data are consistent with the idea that auditory amplification/enrichment could only mitigate cochlear damage by slowing down its degenerative processes when some normal hair cells remained [26].

### 4.2. Amplification and Endbulb Changes

Endbulbs of Held are large and structurally distinct entities within AVCN. Endbulb development begins as a small terminal growth cone with filipodia that grows into a club-shaped swelling in neonatal life and transforms into its mature form by branching repeatedly into a highly arborized structure by 12 weeks of age [41]. In the normal hearing mouse, a mature endbulb is complex in shape, well elaborated, and has many swellings.

Endbulbs undergo significant atrophy with age-related hearing loss; they become devoid of their usual complex branching and their target cells shrink in size following the disruption of acoustic input. Since endbulbs are malleable and exhibit activity-dependent plasticity [45], they offer a good model for investigating the effect of sensory deprivation and sensory stimulation on brain structure and function. The significance of neural activity on the central auditory system has been repeatedly documented (e.g., [67,68]). The consequence of loss of acoustic input is decreased neuronal activity. Prolonged sensory deprivation is known to reduce the number of functional presynaptic terminals in the brain [69]. Maintenance of sound driven activity in the endbulb is pivotal to ensuring high fidelity transmission of acoustic information [43]. Active endbulbs exhibit larger terminal swellings and more release sites than inactive endbulbs [43,45,70], which is consistent with the “synaptic size principle” which basically states that the larger a synapse, the stronger its effect [71,72].

Our data suggests that acoustic amplification maintains the morphology and complexity of endbulbs particularly where there is hearing loss; sound enriched subjects exhibited larger terminal swellings, suggesting an activity-dependent trophic effect. Similar to what has been reported [41], we observed the complex endbulb morphology in young mice before progressive hearing loss had commenced. As hearing loss advanced, however, endbulb structures gradually lost their elaborate appearance. In the 52-week-old DBA/2 mice, endbulb atrophy became severe such that the tiny, nodular swellings were conspicuously absent. On the other hand, stimulation of all mice using the amplified environment bolstered the endbulbs when compared to those that were not treated. Evidence from quantitative analysis revealed that the surface area, volume, and shape factor of the endbulb was preserved in the treated subjects, implying that they maintained their structural complexity and functional contact with the target neurons. We infer that the normally large and elaborated endbulbs suggest the availability of more functional release sites and an increase in the efficiency of synaptic transmission, which would ultimately enhance the synaptic strength of this pathway. This pattern of change was reported when Webster [28] induced a conductive hearing loss to neonatal CBA/J mice at postnatal day 4 (P4). The author then provided 34 dB of amplified sound to the experimental mice from P11-P24 in one group and continuously from P11 to another group until they were sacrificed at P45. Their result showed that the mice that were deprived of continuous amplified sound exhibited a significantly reduced cochlear nucleus volume as compared to those that received amplified sound, suggesting that sound restoration could halt further deterioration of auditory structure.

### 4.3. Early Versus Late Treatment and Critical Period

The brain displays a dramatic capacity to change in response to environmental manipulation. Prior literature has discussed that the malleability of the brain is most remarkable at a young age [23,57,73,74]. Thus, it is of interest to note in this study that the most significant effect of acoustic enrichment was seen in the early age cohorts, and it appears that this effect waned as treatment was delayed in the late enrolled groups. It remains unclear why the outcome waned with time and age. Does it have to do with the critical period or because we were dealing with more mature brains that could be less malleable and plastic? For instance, hearing loss progressed rapidly as age advanced in DBA/2 mice, but amplification appears not to have any effect on the older endbulb. But it was not clear if it was due to age or late intervention. On the contrary, endbulb changes in the C57Bl/6 became more noticeable as hearing loss worsened with age.

Available evidence suggests that the adult brain is plastic [75]. So, the question is whether the lack of an effect in the DBA/2 mice enrolled at 12 weeks was due to late intervention or nonplasticity. To exclude the plausible nonplasticity notion, another group of mice was employed at 4 weeks of age and exposed to sound amplification up to 20 weeks of age. Interestingly, there was a significant difference between the control and sound enriched endbulbs, as the enriched endbulbs were larger and showed more complex endings. The reason for this observation may not be far-fetched. On one hand, in the late treatment group, the loss of peripheral auditory receptors resulting from hair cell loss may have precluded the sound from getting to the brain [76]. It can be argued that the early treated cohorts were also losing their auditory receptors. We think robust sound amplification was able to compensate for the missing receptors. On the other hand, due to the time between longstanding hearing loss and commencement of sound augmentation, hearing loss might have occurred that compromised treatment.

Studies have shown that disrupted cortical organization [24,77] may be reversed by exposure to tones when presented while the animal was still young and within its “plastic” critical period [57]. Evidence from human studies also shows that it is early cochlear implantation of children with hearing loss and deafness that yields the best outcome [78]. With the phenomenon of brain plasticity, adult animals and humans can learn new skills and change their behaviors. It seems rehabilitation of hearing function in the adult may be possible if the period between the hearing loss and the treatment intervention is not too long.

### 4.4. Impact of Hearing Loss and Ageing on Endbulb Morphology

In our mouse models of hearing loss—DBA/2 and C57Bl/6—we had consistently observed that endbulb surface area and volume decreased with the progression of hearing loss and ageing in both the treated and untreated mice (Figure 7, Figure 11 and Figure S5). Incidentally, mice with long durations of hearing loss suffered more severe endbulb atrophy, which made it difficult to get a clear picture of structural dynamics with the extra amplification. We previously showed that endbulb volume and surface area decrease progressively in ageing normal hearing CBA/CaH mice, even though these mice had no elevation of hearing threshold [79]. What is clear, however, is that stimulation has an enhancing impact on endbulbs that receive early amplification treatment (e.g., termination dates of 12, 14, and 16 weeks of age).

### 4.5. Optimal Time to Fit Hearing Aids

Is there a critical period for fitting hearing aids and is timing likely to affect its outcome? According to our data, the time to fit hearing aids is when there are functional hair cells. Our results from the C57Bl/6 strain suggest that the endbulb is still plastic at 52 weeks, meaning that the aged brain might still be plastic [54]. As many frequencies were represented at the time the intervention was initiated, the outcome was better. Therefore, hair cells are needed to get sound into the brain. Hearing aids do not stop the genetically determined loss of hair cell receptors, which undoubtedly accounts for why they often fail to meet some patients’ expectations. The variability surrounding the efficacy of hearing aids reflects how much more we need to learn about hearing loss and hearing rehabilitation.

What might be the outcome if the mouse models of progressing hearing loss (DBA/2, C57Bl6) were implanted with cochlear implants? Our experiment did not investigate how electrical stimulation via cochlear implants might influence auditory plasticity in mice (DBA/2) with early onset of hearing loss. However, there are many reasons to expect that cochlear implants would have provided positive results in terms of hearing thresholds and endbulb preservation. Our lab had previously shown that cochlear implantation restored auditory synapse pathology in cats [19], a result that provided physical evidence for their efficacy. Congenitally deaf cats were stimulated for 3 months with a six-channel cochlear implant. This device used human speech-processing programs. After the experiment, there was restoration of auditory nerve synapses in cats following cochlear implantations and cats responded to environmental sounds.

In humans, age and duration of usage of hearing aids are the primary determinants for satisfaction [80]. A negative perception was reported in the elderly, whereas positive satisfaction has been reported among the frequent users of hearing aid devices [80,81]. We know that structure is related to function; pathologic endbulbs may distort the temporal representation of the sound that is conveyed to the target bushy cell [42]. We think the issue in the treatment of hearing loss is not limited to audibility alone but also include features of sound perception with respect to timing, location in space, presence of background noise, and binaural fusion of frequencies.

Unfortunately, by the time many patients with hearing loss decide to see a clinician or an audiologist, significant atrophy may have already occurred throughout the auditory brain. Furthermore, conventional hearing tests may not reveal this loss [82,83]. It remains a challenge to treat a compromised brain, which means clinicians are advised to use multiple non-invasive audiometric tests when assessing candidates in order to avoid missing the symptoms of hidden hearing loss.

## 5. Conclusions

The results of this study represent an important new contribution to our understanding of changes in the brain with progressive hearing loss and how it changes with stimulation. Three major ideas emerge with respect to our data: first, delayed treatment of age-related decline in auditory acuity and hearing loss will negatively impact the outcome of hearing aids. Second, early treatment by amplifying sounds to maintain auditory sensation levels (e.g., using hearing aids) will optimize treatment outcomes by delaying brain pathologic changes that accompany hearing loss. Third, by the time there is significant cochlear receptor loss, hearing aids will not be effective. Therefore, regular and thorough hearing screening tests and early treatment of hearing loss would help to limit the disabling symptoms of hearing loss.

## Figures and Tables

**Table 1:** Morphological measurements of endbulbs of Held by age and treatment for DBA/2 mice.

	Age @ Enrollment (Weeks)	Age @ Termination (Weeks)	N	Surface Area (µm^2^)	Volume (µm^3^)	SA/Vol Ratio	Shape Factor
Control	4	12	75	187.0 ± 127.0	40.8 ± 25.9	4.61 ± 1.11	5.78 ± 2.74
	6	14	44	169.7 ± 119.3	40.7 ± 24.6	4.07 ± 0.74	4.84 ± 2.32
	8	16	25	137.5 ± 48.6	35.1 ± 12.1	3.94 ± 0.61	4.31 ± 1.13
	12	20	23	118.9 ± 74.8	32.6 ± 21.4	3.78 ± 0.54	3.65 ± 1.14
	4	20	63	115.0 ± 54.4	29.0 ± 14.7	4.07 ± 0.77	4.00 ± 1.17
	--	52 *	50	99.8 ± 45.5	26.5 ± 12.8	3.86 ± 0.62	3.55 ± 0.97
Amplified	4	12	33	250.9 ± 157.3	50.4 ± 27.6	5.20 ± 1.51	7.46 ± 3.29
	6	14	168	199.8 ± 131.6	47.3 ± 31.2	4.41 ± 1.14	5.62 ± 2.47
	7	16	71	210.8 ± 131.1	48.1 ± 24.6	4.32 ± 1.07	5.88 ± 3.20
	12	20	61	111.2 ± 77.6	31.1 ± 23.5	3.76 ± 0.59	3.53 ± 1.04
	4	20	69	139.9 ± 63.4	34.1 ± 15.1	4.15 ± 0.61	4.53 ± 1.23

A number of endbulbs (N) were collected and analysed in each age/treatment cohort. The asterisk (*) indicates that the cohort was raised in normal animal vivarium and not enrolled in sound stimulation paradigm.

**Table 2:** Morphological measurements of endbulbs of Held by age and treatment for C57Bl/6 mice.

	Age @ Enrollment (Weeks)	Age @ Termination (Weeks)	N	Surface Area (µm^2^)	Volume (µm^3^)	SA/Vol Ratio	Shape Factor
Control	24	32	61	161.5 ± 73.2	46.6 ± 20.5	3.56 ± 0.82	4.20 ± 1.74
	32	40	39	146.5 ± 78.5	39.2 ± 21.6	3.85 ± 0.74	4.22 ± 1.35
	44	52	69	139.2 ± 77.0	36.2 ± 19.7	3.92 ± 0.59	4.21 ± 1.33
Amplified	24	32	38	177.9 ± 85.3	49.9 ± 25.9	3.69 ± 0.79	4.55 ± 1.76
	32	40	70	183.1 ± 89.9	49.8 ± 25.0	3.78 ± 0.71	4.68 ± 1.42
	44	52	115	157.9 ± 76.9	44.2 ± 21.3	3.64 ± 0.65	4.22 ± 1.31

N = total number of endbulbs analysed in each age/treatment cohort.

## Data Availability

The original contributions presented in this study are included in the article/Supplementary Material. Further inquiries can be directed to the corresponding author(s).

## Supplementary Materials

The following supporting information can be downloaded at: https://www.mdpi.com/article/10.3390/brainsci15080888/s1, Figure S1. Sound stimulation and amplification paradigm. Figure S2. Endbulb of Held from a young control mouse. Imaris software calculated EB surface area and volume. Figure S3. Progression of hearing loss in untreated C57Bl/6 mice as revealed by increases in hearing thresholds. Figure S4. Examples of endbulbs from untreated DBA/2 mice that were raised in the animal vivarium until 52 weeks of age. Note the simplified structure and general lack of tertiary branches as compared to a “normal control” endbulb shown in S2. Figure S5. Longitudinal summary of endbulb data for all cohorts by age. Note that the gains resulting from the enriched auditory environment were not lasting.
