# Supplementary material for: Effect of Sound Amplification on Central Auditory Plasticity: Endbulb of Held as a Substrate
Source: Brain Sci. 2025 Aug 20;15(8):888. doi: 10.3390/brainsci15080888 (PMC12384917; doi:10.3390/brainsci15080888)
Supplement: Supplementary file 1 [file brainsci-15-00888-s001.zip › Supplementary legend.pdf]

**Supplementary Figure S1.** *Sound stimulation and amplification paradigm.* **A.** The stimulation parameters, sound environment, and weekly audiogram of an individual experimental subject are shown. Sound amplification was tailored to the frequency-specific hearing loss profile of each subject using the recorded baseline hearing threshold as a reference for amplification. **B.** A 20-sec. sample spectrogram shows the varying frequency content of recorded sounds (mouse, cat, dog, and human sounds, street noises, animal room noises, and artificial dynamic ripple stimuli). These sounds are within the normal hearing range of mice.

**Supplementary Figure S2**. *Endbulb of Held from a young control mouse. Imaris software calculated EB surface area and volume*. The portion of the auditory nerve distal to the endbulb is cut off (white), and the surface area and volume of each endbulb was quantified.

**Supplementary Figure S3.** *Progression of hearing loss in untreated C57Bl/6 mice as revealed by increases in hearing thresholds*. Mean ABR thresholds are shown for all ages and frequencies at which C57Bl/6 mice were examined. Animals were enrolled as un-amplified controls in the sound stimulation paradigm (colors). The late-onset age-related progression of hearing loss is evident, particularly for mid-frequencies (8-16 kHz) beginning around 34-weeks-of-age. Mean thresholds for age-matched CBA mice (grey) are provided as reference.

**Supplementary Figure S4.** *Examples of endbulbs from untreated DBA/2 mice that were raised in the animal vivarium until 52 weeks of age.* Note the simplified structure and general lack of tertiary branches as compared to a "normal control" endbulb shown in S2.

**Supplementary Figure S5.** *Longitudinal summary of endbulb data for all cohorts by age. Note that the gains resulting from the enriched auditory environment was not lasting.* Data from normal-hearing CBA/Ca, early-onset hearing loss DBA/2 and late-onset hearing loss C57Bl/6 mice are plotted according to age to show how the different strains compare across time and treatment. The plots emphasize comparisons between control (unamplified) and experimental (amplified) groups and show that amplified stimulation definitely delays the rate of endbulb atrophy. Endbulb surface area tended to be similar between normal-hearing CBA/Ca mice and untreated controls of both strains. Animals receiving amplification exhibited larger endbulbs. The volume of endbulbs belonging to CBA/Ca mice was usually between that of treated and untreated mice. Endbulbs from normal-hearing CBA/Ca mice consistently had lower surface area to volume ratios than both DBA/2 and C57Bl/6 mice, regardless of treatment condition. Shape factor measurements for CBA/Ca mice were also

lower than what was observed in other strains, although they tended to align with untreated cohorts more closely.
