# Supplementary figures and images for: Effect of Sound Amplification on Central Auditory Plasticity: Endbulb of Held as a Substrate
Source: Brain Sci. 2025 Aug 20;15(8):888. doi: 10.3390/brainsci15080888 (PMC12384917; doi:10.3390/brainsci15080888)

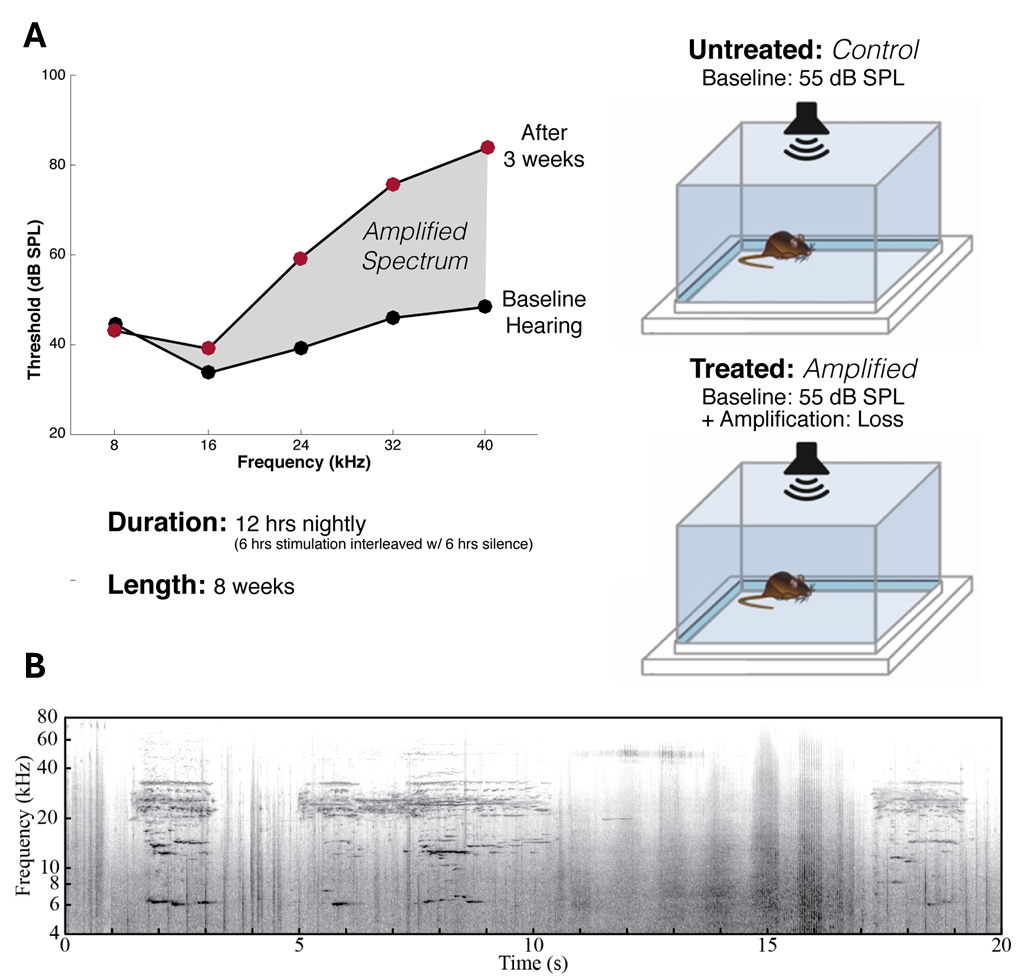

Supplement: Supplementary file 1 [file brainsci-15-00888-s001.zip › Supplementary Figure S1.tif]

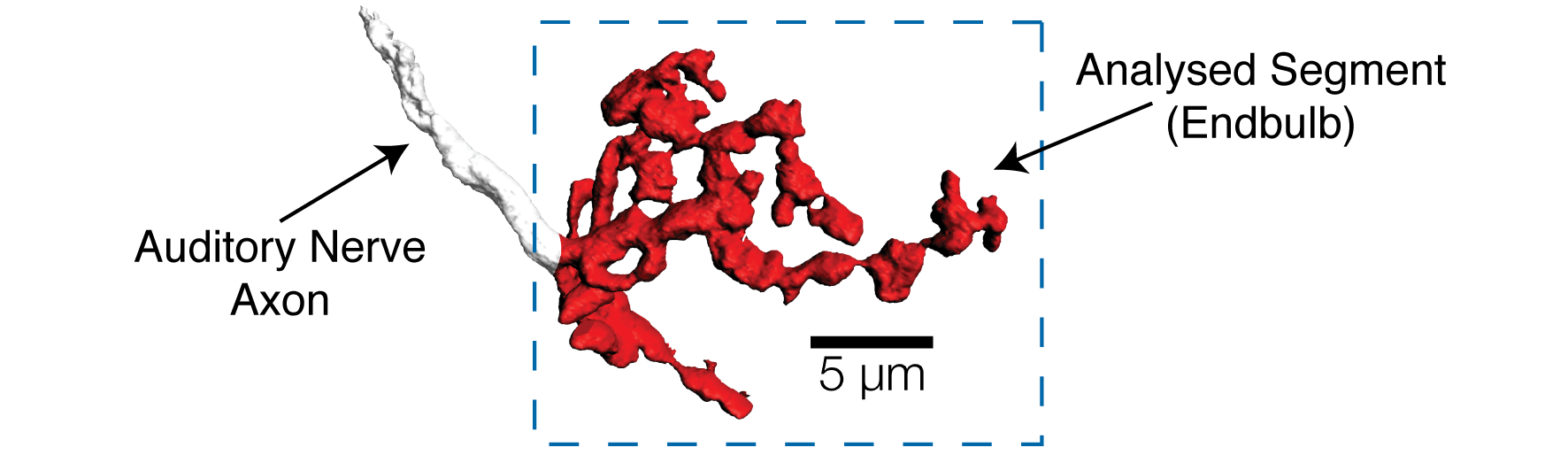

Supplement: Supplementary file 1 [file brainsci-15-00888-s001.zip › Supplementary Figure S2.tif]

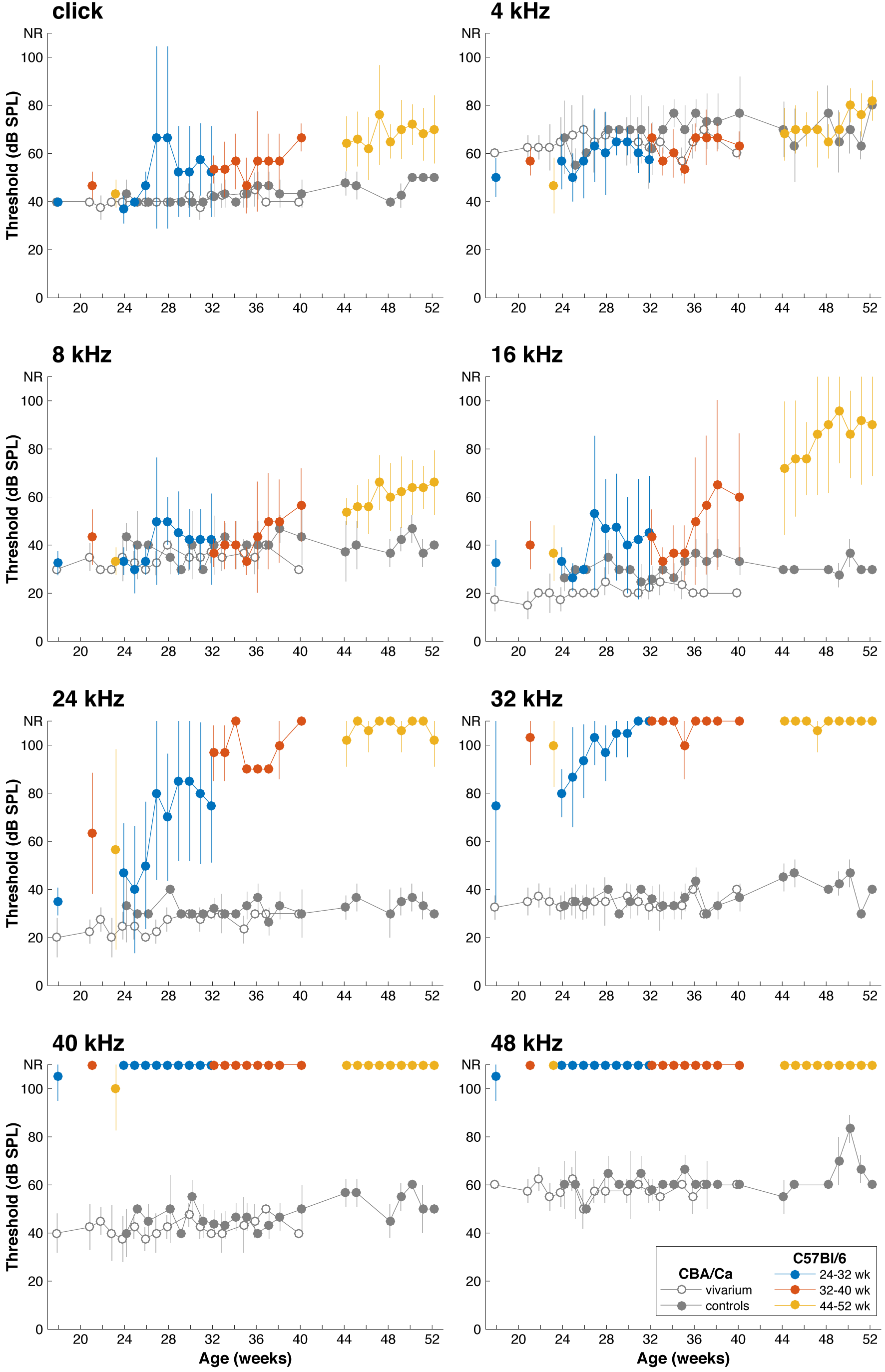

Supplement: Supplementary file 1 [file brainsci-15-00888-s001.zip › Supplementary Figure S3.tif]

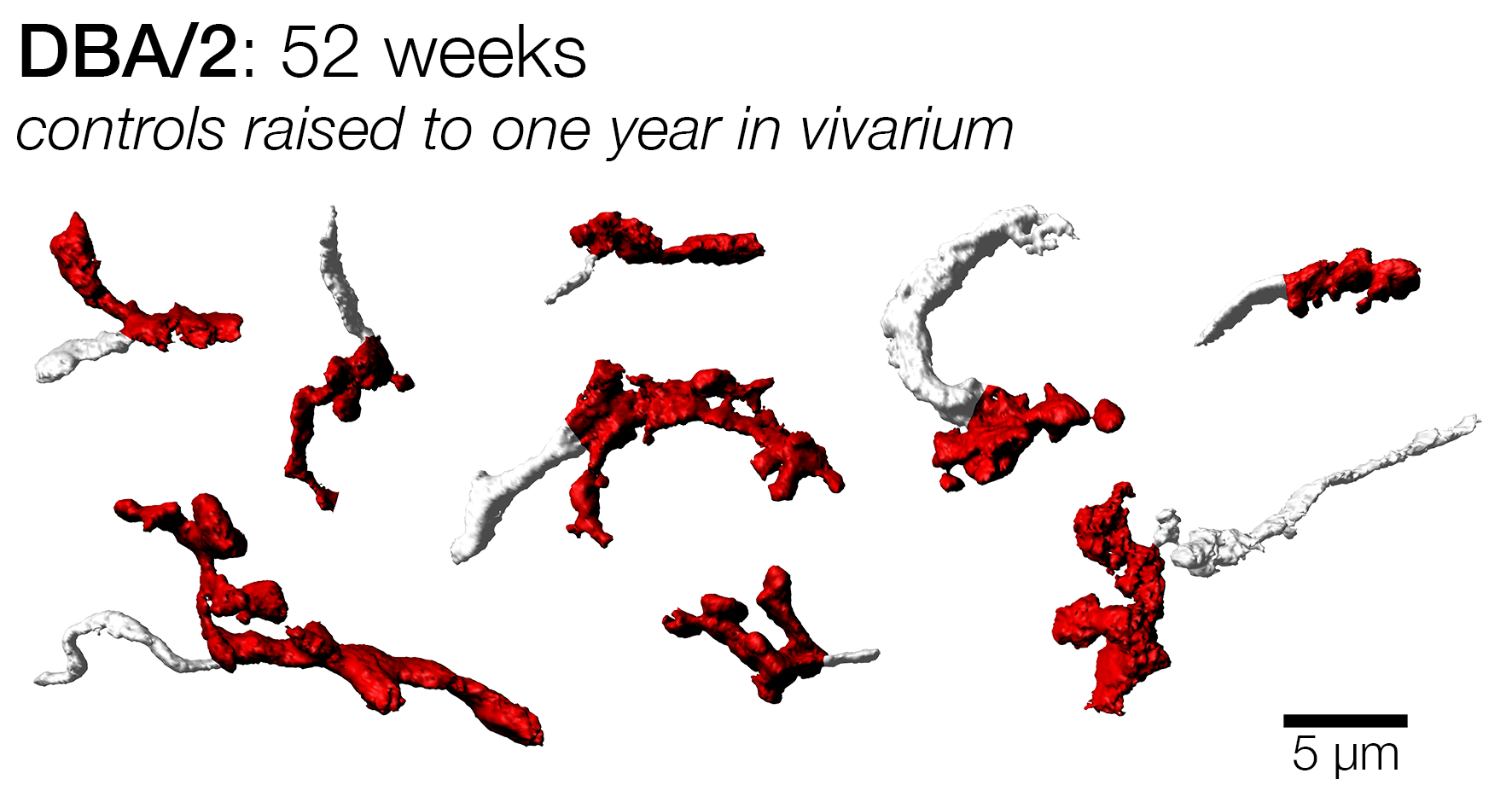

Supplement: Supplementary file 1 [file brainsci-15-00888-s001.zip › Supplementary Figure S4.tif]

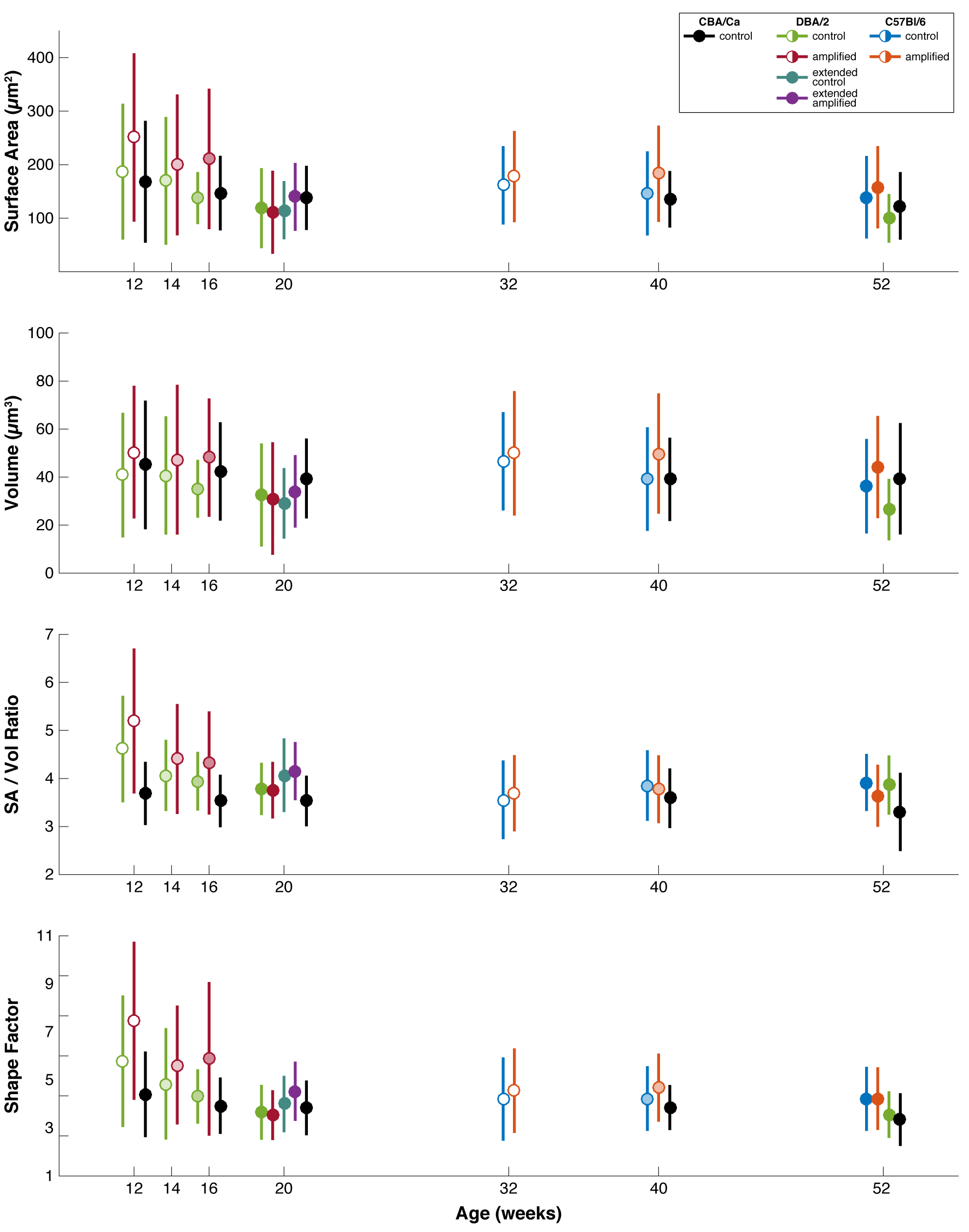

Supplement: Supplementary file 1 [file brainsci-15-00888-s001.zip › Supplementary Figure S5.tif]
